# Draf III frontal sinus surgery for the treatment of Pott’s puffy tumour in adults: our case series and a review of frontal sinus anatomy risk factors

**DOI:** 10.1007/s00405-020-05980-2

**Published:** 2020-04-29

**Authors:** Alfonso Luca Pendolino, Foteini Stefania Koumpa, Henry Zhang, Samuel C. Leong, Peter J. Andrews

**Affiliations:** 1 Department of ENT, Royal National Ent and Eastman Dental Hospitals, 47-49 Huntley St Bloomsbury, London, WC1E 6DG UK; 2 Ear Institute, UCL, London, UK; 3 Skull Base Unit, Department of Otorhinolaryngology-Head and Neck Surgery, Aintree University Hospital NHS Foundation Trust, Liverpool, UK

**Keywords:** Pott’s puffy tumour, Frontal sinus, Frontal sinusitis, Endoscopy

## Abstract

**Purpose:**

We present our case series of four adult patients with Pott’s puffy tumour (PPT), successfully treated with Draf III over a mean period of 11 months. A critical review of the literature is also provided.

**Methods:**

A retrospective review of patients undergoing Draf III for PPT from January 2018 to January 2019 was performed.

**Results:**

Four consecutive male patients ranging from 26 to 62 years, with a mean age of 49.5 ± 16.3 years, undergoing Draf III for Pott’s puffy tumour were included. Two patients had a Kuhn type IV frontal cell narrowing the frontonasal pathway and presented without previous sinus surgery, whereas the other two had previous sinus surgery. The success rate of the operation was 100% with an average length of follow-up of 11 months (range 5–18).

**Conclusion:**

In our experience, the Draf III procedure is a highly effective treatment of PPT. In particular, we have demonstrated it to be very effective in accessing highly positioned Kuhn type IV cells.

## Introduction

Pott’s puffy tumour (PPT) is a subperiosteal abscess of the anterior frontal plate associated with osteomyelitis. It was first described by Sir Percivall Pott in 1775 as a complication of a frontal sinusitis [1]. It is a relatively rare but serious condition usually presenting as a localized swelling of the forehead, and can be associated with periorbital oedema, purulent nasal discharge, fever, headache and vomiting. Occasionally, it may cause severe intracranial complications like meningitis, epidural abscess, subdural empyema, intracerebral abscess, and dural sinus thrombophlebitis as a result of the spreading of the infection through bony erosions or septic thrombosis [2]. The most frequently affected age group is adolescents, while its occurrence in the adults is considered rare [3–5].

Treatment of PPT and prevention of its complications involves a combination of intravenous antibiotics and surgical drainage. Empiric antibiotic therapy consists of broad-spectrum antibiotics with good penetration to central nervous system, commonly clindamycin, ceftriaxone, metronidazole and vancomycin, and it must be started as soon as possible. Then, the antibiotic can be changed accordingly to the results of culture and microbial susceptibility testing, and should be prolonged for at least 6–8 weeks postoperatively [6–8].

However, surgical treatment remains the keystone in the management of these patients and may vary from external surgical approach alone, endoscopic sinus surgery (ESS) either alone or in combination with external drainage. External approach to the frontal sinus is easy and rapid, and it allows a direct visualization of the sinus. Nonetheless, this approach is associated with facial scars and does not address the frontal sinus blockage causation which theoretically may lead to a relapse of the abscess. ESS approach as well as addressing the site of frontal sinus blockage also prevents the need for an external facial scar [4]. The extend of ESS may vary from a simple (Draf I) to an extended drainage of the frontal sinus (Draf II), with the latter achieved by resecting the floor of the frontal sinus between the lamina papyracea and the middle turbinate (Draf IIa) or between the lamina papyracea and nasal septum (Draf IIb). In some cases, an endonasal median drainage (Draf III or endoscopic modified Lothrop procedure) is required to achieve the maximum possible opening [9]. The challenge whilst addressing the frontal sinus blockage caused by a Kuhn type IV cell [10] is whether endoscopically it can be reached and cleared properly. Evidence in the literature demonstrating the long-term outcomes of Draf III for the surgical treatment of PPT is poor and whether Draf III can be used in all cases, especially in those with frontal sinus obstructing cells, has not been reported [11]. We present our case series of four adult patients with PPT successfully treated with Draf III over a mean period of 11 months, including two with highly placed obstructing Kuhn type IV cells.

## Materials and methods

A retrospective review of patients undergoing Draf III for PPT from January 2018 to January 2019 at the Royal National Throat Nose and Ear Hospital, London, United Kingdom was performed. The diagnosis was confirmed by clinical examination and imaging (CT and MRI scan). Population data including clinical presentation, imaging, surgical operative reports final outcomes, interval between diagnosis and treatment, pre- and post-operative medical treatment, length of follow-up were recorded and analysed. Formal ethical approval was not required because all the data were processed through local hospital trust audit policy. All investigations and treatments were carried out in line with accepted clinical practice.

### Surgical technique

Draf III, also called endonasal median drainage or modified endoscopic Lothrop procedure, was performed under general anaesthesia and with image guidance assistance. In this procedure, a wide opening between the two frontal sinuses and the nasal cavity is achieved. Once an opening of frontal sinus between lamina papyracea and nasal septum (Draf IIb) has been performed bilaterally, the frontal sinus floor in front of the olfactory cleft and the intersinus septum is removed. The middle turbinate is resected very gently from an anterior to posterior direction, along its origin at the skull base. A diamond burr drill is used to reduce the frontal beak and the frontal intersinus septum or septa, if more than one is present [9]. Finally, the nose is packed with varnished whitehead ribbon gauze or absorbable packs soaked in betamethasone 0.1% drops to reduce scarring and post-operative oedema.

## Results

### Population

Four consecutive male patients ranging from 26 to 62 years, with a mean age of 49.5 ± 16.3 years, undergoing Draf III for PPT were included. Clinical and demographic data are summarized in Table 1.

**Table 1:** Patients’ clinical and demographic data

	Age	Sex	History and presentation	Pathology	Comorbidities	Previous sinonasal operations	Intracranial findings	Frontal sinus anatomy	Surgery for PPT	Microbiology	Interval from surgery to post-operative CT scan (months)	Follow up (months)
1	26	M	Swelling forehead in the past 4 months, headache	CRSwNP	None	1 × ESS and polypectomy	Meningeal enhancement on MRI (no intracranial extension)	None	1. 4 × revision ESS (elsewhere) 2. Draf III (5 years later)	Negative	7 and 13^a^	13
2	59	M	Swelling forehead in the past 8 months, headache, loss of sense of smell, nasal blockage	CRSsNP	Diabetes	None	None	Kuhn type IV cell	1. Draf III	Negative	9	9
3	51	M	Swelling forehead in the past 26 months, headache, peri-orbital cellulitis, blurred vision	CRSsNP	HIV, Psoriatic arthritis	2 × ESS	Skull erosion (previous subdural empyema in 2015)	None	1. ESS (elsewhere) 2. Draf III (2 years later)	Negative	8 and 18^a^	18
4	62	M	Swelling forehead in the past 11 months, headache	CRSsNP	None	None	None	Kuhn type IV cell	1. ESS + external drainage 2. Draf III (8 months later)	*Streptococcus* *Milieri*	3	5

*PPT* Pott’s puffy tumour, *ESS* endoscopic sinus surgery, *CRSwNP* chronic rhinosinusitis with nasal polyps, *CRSsNP* chronic rhinosinusitis without nasal polyps

^a^Patient 1 and 3 received short- and long-term post-operative CT scans before discharging

### Clinical presentation and history

All patients presented to the Emergency Department with a forehead swelling and headache overlying the frontal sinuses. All but one had a history of chronic rhinosinusitis (CRS) without nasal polyps with only one patient having nasal polyps under medical treatment. Two patients had a history of previous sinonasal surgery for their CRS. None of them had intracranial complications at presentation. All patients received broad spectrum intravenous antibiotic with good penetration to the central nervous system before undergoing Draf III. Two patients had already had ESS with simple frontal sinus drainage (Draf I) with or without external drainage. Unfortunately, PTT recurred in all of them and they underwent Draf III under our care. In another patient, Draf III was chosen as the first approach from the beginning based on the presence of an unfavourable frontal sinus anatomy at the CT scan.

### Imaging

**Fig. 1:**
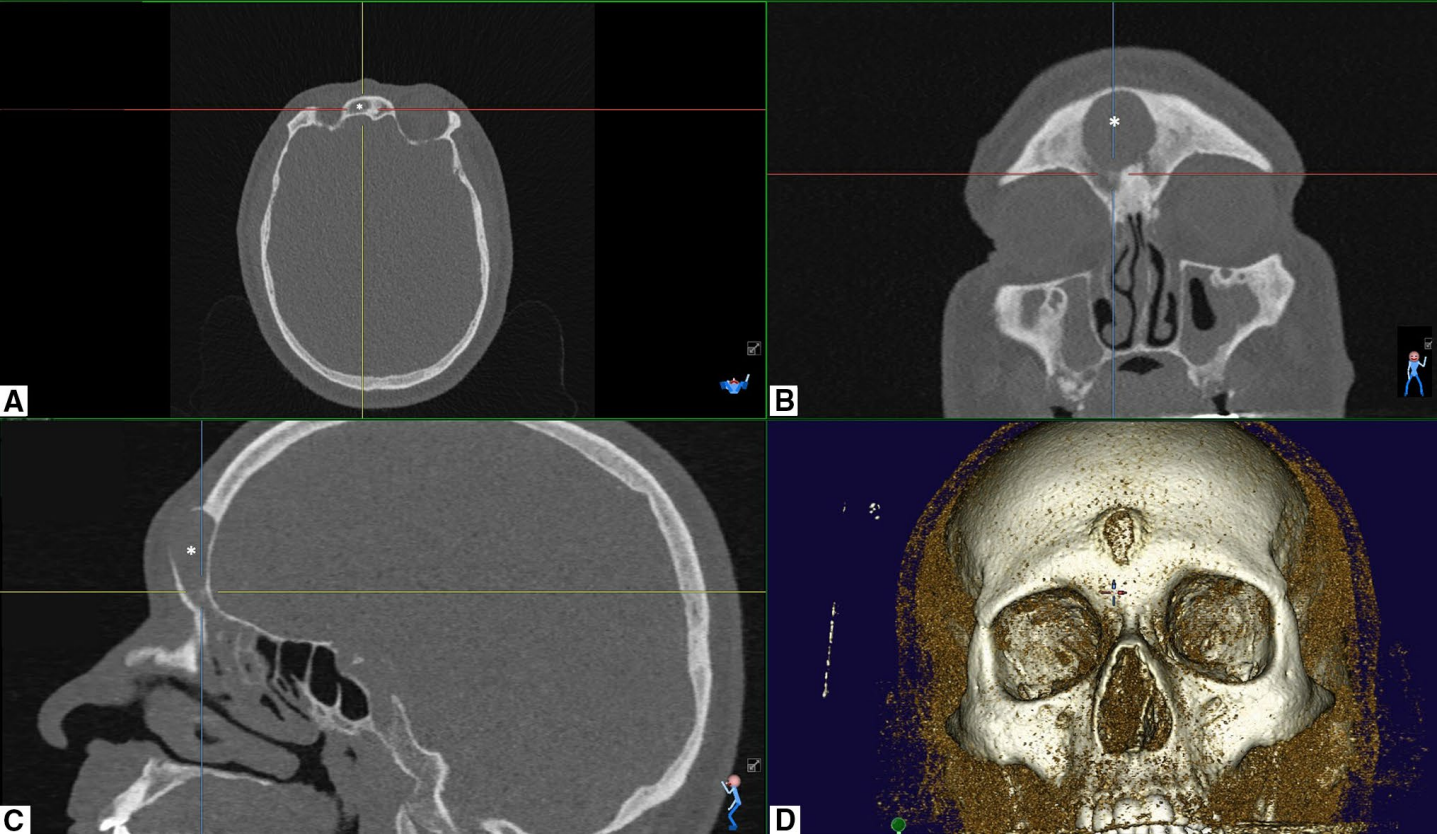
Pre-operative CT scan of the 59-year-old male presenting with Pott’s puffy tumour. Please note the Kuhn type IV cell (*). **a** Axial, **b** coronal and **c** sagittal view. **d** 3D reconstruction

**Fig. 2:**
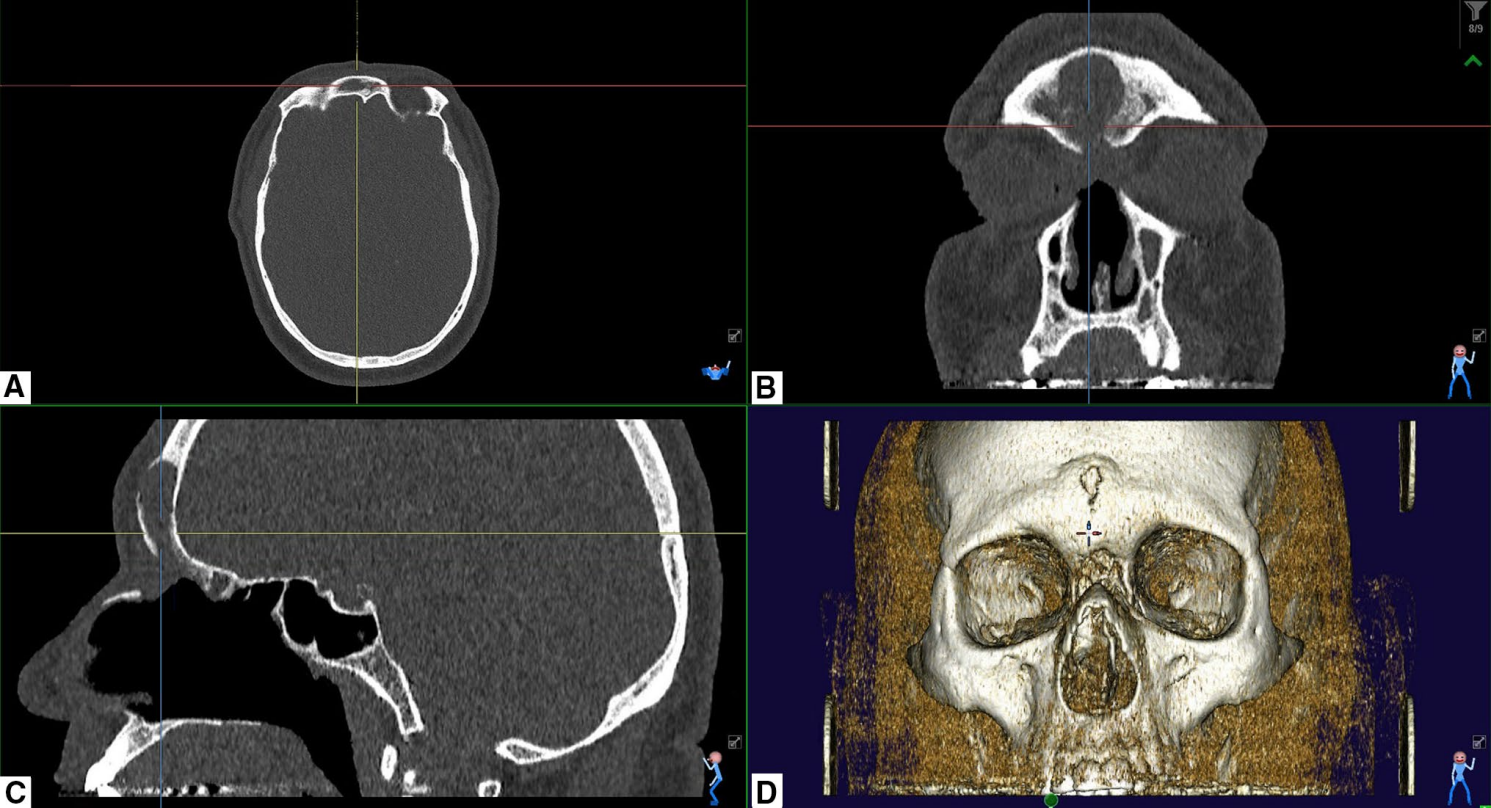
Post-operative CT scan of the 59-year-old male, 9 months after Draf III. Kuhn type IV cell has been opened and is not visible at the CT scan. There is residual inflammation in the frontal sinus which is compatible with the underlying chronic rhinosinusitis. A wider sinonasal frontal cavity has been obtained. **a** Axial, **b** coronal and **c** sagittal view. **d** 3D reconstruction

**Fig. 3:**
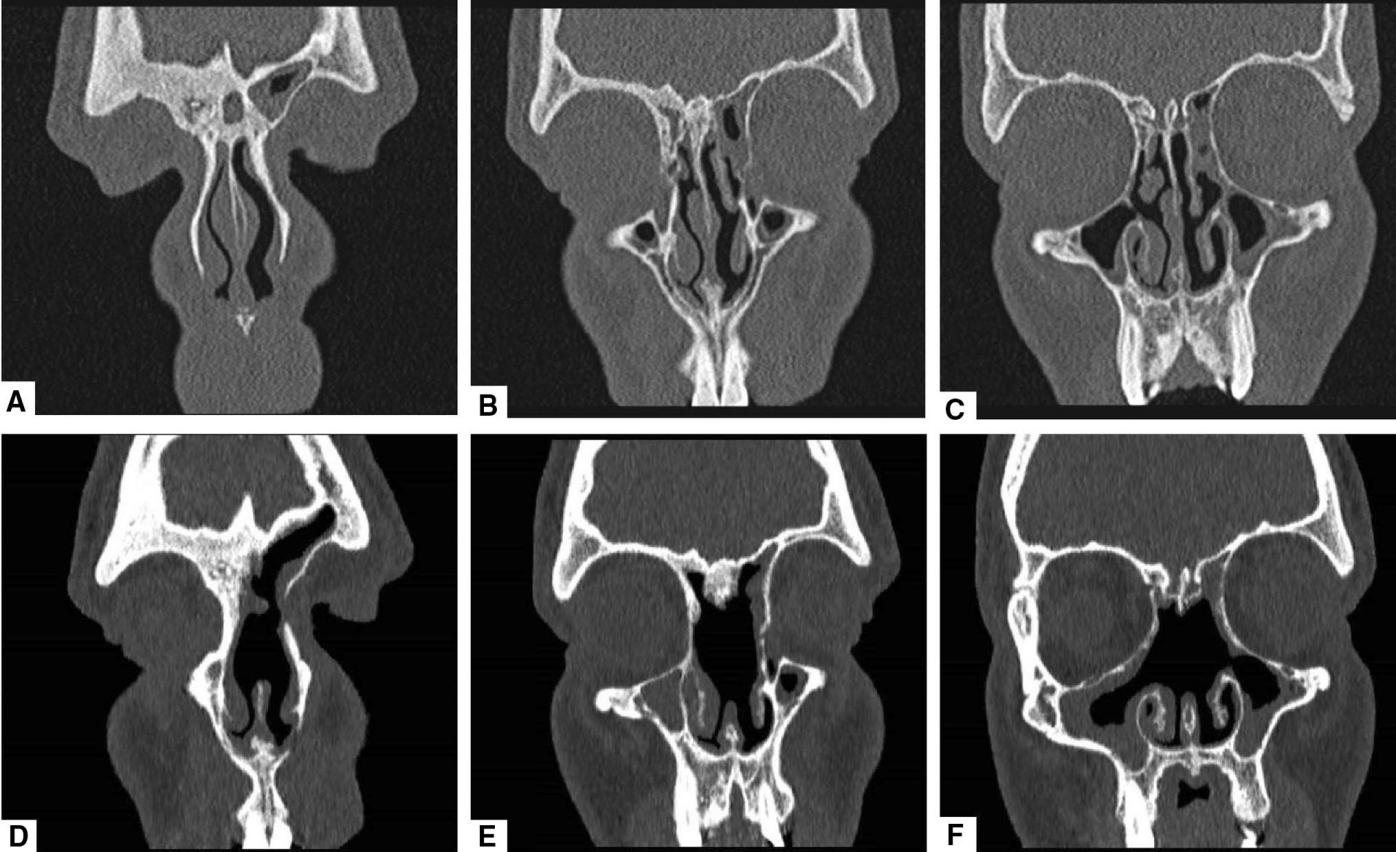
**a–c** Sequential slices in coronal view of the pre-operative CT scan of the 26-year-old male presenting with pott’s puffy tumour. Please note the osteitis of the frontal bone blocking the frontonasal pathway as a result of the previous endoscopic sinus surgeries and the ongoing inflammation. **d–f** Sequential slices in coronal view of the post-operative CT scan 13 months after Draf III

PTT was diagnosed preoperatively by means of CT and MRI scans. In one patient, the CT scan showed an asymptomatic meningeal enhancement. Two patients had a Kuhn type IV cell obstructing the frontal recess (Fig. 1). CT scan was performed post-operatively in all the patients to rule out any intracranial complication, stenosis or recurrence (Table 1, Figs. 2, 3). Patients two and four were lost earlier at follow-up, while the other two patients had a longer follow-up and received a second post-operative CT scan to evaluate long-term results (Table 1).

### Outcomes

Draf III was successful in all cases. There were no acute complications. Histology of the specimens showed inflammatory mucosa in all cases. Microbiology culture was positive for *Streptococcus milleri* in one case while showed normal flora in the rests. All patients received broad-spectrum antibiotics with good central nervous system penetration postoperatively for at least 2 weeks and adjusted according to microbiology findings. In our experience, following Draf III surgery, patients benefit from a long-term course of macrolide treatment for at least a further 8 weeks (clarithromycin 500 mg daily for 8 weeks). Patients are started on a short course of oral steroid (prednisolone 40 mg daily for 5 days), if not contraindicated. Nasal douches with normal saline are highly recommended at least twice a day followed by intranasal steroid drops (bethamethasone sodium phosphate 0.1%, 2 drops each nostril twice a day) in the “Kaiteki” position were also suggested for 4 weeks. At 4 weeks, patients are debrided in out-patients under local anaesthetic whereby crusts and debris are removed. Out of the four patients, only one required endoscopic nasal de-crusting under general anaesthesia 2 weeks after the operation. One patient developed sinusitis within the first month postoperatively, which eventually resolved with oral antibiotics. Once the cavity starts stabilising at 4 weeks, patients continue with douching and commence Fluticasone propionate 400 μg nasal drops (400 μg divided between the nostrils) twice a day.

### Follow-up

The success rate of the operation was 100% with an average length of follow-up of 11 months (range 5–18). All patients were followed up with nasendoscopy in an outpatient setting and none of them required revision surgery for recurrence.

## Discussion

PPT most frequently occurs in paediatric and adolescent populations while it is considered rare in adults [12]. In young people, PPT is considered more prevalent as a result from either an anatomically undeveloped frontal sinus or an increased blood flow within the diploic veins found in adolescence [13, 14]. In the adults, the main cause of PPT is stenosis of the frontoethmoidal duct, as a result of recurrent sinusitis or head surgery/trauma [14, 15]. CRS is considered as a risk factor for PPT development [16, 17], and all of our patients had a history of CRS with or without nasal polyps. It has also been speculated that the status of the host and his past history are important factors in adult cases. Underlying diseases such as diabetes, chronic renal failure, and aplastic anaemia have been reported to be developing risk factors but not clear exacerbating factors of PPT. Intranasal cocaine use has also been described as a predisposing factor related to its ability to compromise bones and generate intranasal inflammation [14]. A male to female preponderance has also been reported [3, 18] and our study strongly confirms this with all four cases being male.

In our cohort of four patients, we identified two main causes for PPT. The first cause appears to be frontal sinus ostium stenosis following previous sinus surgery which has been already described in the literature as a risk factor for PPT development. Repeated frontal sinus surgery, in fact, can cause a bony remodelling with a potential narrowing of the frontonasal duct (Fig. 3). The second cause was the presence of a highly placed Kuhn type IV cell resulting in frontal sinus obstruction which we found in two of our cases (Fig. 1). Kuhn type IV cells are single, but isolated cells found within the frontal sinus [10, 19], which can be found with a frequency ranging from 1.3% to 8.5% [10, 20]. The origin of the isolated frontal cell (Kuhn type IV cell) remains unclear and updated frontal sinus classification systems postulate it to be related to the midline intersinus septum [21]. The presence of frontal cells, especially of a Kuhn type III or IV cell, has been reported to correlate with a significantly higher incidence of frontal sinus disease [22] as well as frontal sinus revision surgery [23]. However, to our knowledge, a Kuhn type IV cell has never been described as a risk factor for PPT development. In our opinion, this type of cell, even if rarely found, can create a higher and distal block of the sinonasal pathway with an increased risk of frontal sinusitis and consequent potential complications. This risk becomes even higher if one or more additional risk factors of the above-mentioned are present.

Until recently, the surgical treatment of PPT involved many techniques and predominantly an external approach. In a recent review of the literature which included 83 paediatric and adolescent patients surgically treated for PPT, Koltsidopoulos et al., reported that an external approach was used in most of the cases (46.5%), while an endoscopic treatment was used alone in 20% of cases and in combination with an external drainage in 27% of cases. In the remaining cases (3.5%), no surgical treatment was adopted [24]. In another review of the literature published in 2012 including 32 adult patients with PPT after 1990, Akiyama and colleagues reported that external surgical procedure was chosen in 58.1% of the cases, but ESS in combination with an external subperiosteal abscess drainage was adopted in 32.9% of the rest. Only forehead drainage treatment without radical drainage surgery for frontal sinuses was performed in three cases (9.7%). Recurrence was observed in two of the patients undergoing sinus surgery [14]. In 2017, Şimşek reported one case of PPT treated by means of an external approach to drain the abscess and remove the osteomyelitic bone combined with an endoscopic enlargement of the frontonasal duct [25]. Similarly Geyton and colleague performed ESS with external drainage of frontal sinus abscess in a 45-year-old man presented with PPT [26], while Tatsumi et al., performed successfully ESS alone, even if they did not report the extent of the sinus surgery [27]. More recently, another two cases have been reported by two different authors. However, both combined endoscopic and external surgical approach with drainage of the abscess and debridement of necrotic material [5, 28].

Evidence is not available to determine the extent of endoscopic sinus surgery required to prevent recurrence of PPT. Van der Poel et al., in their case series of six paediatric patients successfully used Draf IIa in five cases even if they reported a high rate of re-stenosis with two patients undergoing a Draf IIa revision and another one requiring a Draf III to treat a mucocele [4].

Current indications for a Draf III procedure include; persistent chronic frontal sinusitis with failure of endoscopic frontal surgery, frontal sinus mucoceles, inverted papilloma, osteoma and trauma [29]. In our experience, Draf III should be also considered the procedure of choice in patients with PPT including those with an isolated frontal sinus obstructing cell (i.e., Kuhn type IV cell).

The issue over post-operative care following Draf III is crucial with the need to treat post-operative crusting. Patients will require endoscopic debridement under local anaesthesia at week 3 and potentially week 6. We recommend frequent nasal douches as well as intranasal steroid drops in the “Kaiteki” position to allow for delivery of the drug to the frontal sinuses [30]. We also suggest long-term macrolide antibiotics for 8 weeks in view of their anti-inflammatory and immune-mediating properties [31], compatible with the antibiogram of the microbiology sent out during the procedure. However, there are cases where this fails according to the literature. Sekine et al., described an immunocompromised case of recurrent PPT after a combination of incisional drainage (× 2) and a Draf III who was eventually treated with a combination of repeat ESS, craniotomy, and frontal sinus reconstruction using an anterolateral thigh flap [32]. Similarly, Leong reported a recurrence in one of his two patients undergoing Draf III for PTT [33]. In those recurrent cases, craniotomy and total removal of the affected tissue should be considered.

The optimum timing for PPT surgery, either external or endoscopic frontal sinus surgery, has not been determined. Akiyama et al., reported no correlation between the time from onset to surgery and the incidence of intracranial complications [14]. In the planning of a Draf III procedure for PPT, we advise early administration of intravenous antibiotic and corticosteroids which may help reducing the inflammatory state and thus diminishing the intraoperative bleeding and the risk of surgical complication.

## Conclusions

In our experience, the Draf III procedure is a highly effective treatment of PPT. In particular, we have demonstrated it to be very effective in accessing highly positioned Kuhn type IV cells. This particular frontal sinus anatomical variant is rare but appeared to be a risk factor in our cohort.
